# Derivatives of 5-Aminolevulinic Acid for Photodynamic Therapy

**Published:** 2007-12-11

**Authors:** Ryan F. Donnelly, Paul A. McCarron, A. David Woolfson

**Affiliations:** School of Pharmacy, Queen’s University Belfast, Medical Biology Centre, 97 Lisburn Road, Belfast BT9 7BL, U.K.

**Keywords:** photodynamic therapy, 5-aminolevulinic acid, derivatives, drug delivery, stability, tissue penetration

## Abstract

Photodynamic therapy (PDT) is a clinical treatment that combines the effects of visible light irradiation with subsequent biochemical events that arise from the presence of a photosensitising drug (possessing no dark toxicity) to cause destruction of selected cells. Today, the most common agent used in dermatological PDT is 5-aminolevulinic acid (ALA). As a result of its hydrophilic character, ALA penetrates skin lesions poorly when applied topically. Its systemic bioavailability is limited and it is known to cause significant side effects when given orally or intravenously. Numerous chemical derivatives of ALA have been synthesised with the aims of either improving topical penetration or enhancing systemic bioavailability, while reducing side effects. *In vitro* cell culture experiments with ALA derivatives have yielded promising results. However, if ALA derivatives are to demonstrate meaningful clinical benefits, a rational approach to topical formulation design is required, along with a systematic study aimed at uncovering the true potential of ALA derivatives in photodynamic therapy. With respect to systemic ALA delivery, more study is required in the developing area of ALA-containing dendrons and dendrimers.

## Introduction

Photodynamic therapy (PDT) is a clinical treatment that combines the effects of visible light irradiation with subsequent biochemical events that arise from the presence of a photosensitising drug (possessing no dark toxicity) to cause destruction of selected cells.^1^ The photosensitiser, when introduced into the body, accumulates in rapidly dividing cells and a measured light dose of appropriate wavelength is then used to irradiate the target tissue.^2^,^3^ This activates the drug through a series of electronic excitations and elicits a series of cytotoxic reactions, which can be dependent on or, less commonly, independent of, the generation of reactive oxygen species.^4^

PDT has progressed considerably from the early application of sunlight and haematoporphyrin derivative, to the use of Photofrin^®^, and to second generation preformed photosensitisers and topical (surface) application of the prodrug, 5-aminolevulinic acid (ALA) which leads to *in situ* synthesis of the potent endogenous photosensitiser protoporphyrin IX^5^ (Daniell and Hill, 1991). PDT is now used for a variety of malignant and pre-malignant skin disorders, as well as certain internal cancers. Topical PDT has been reviewed comprehensively.^6^–^8^ The popularity of ALA, as the most commonly studied agent for PDT, is clearly evident in the number of published articles on the topic, which has increased markedly from 2 in 1991 to about 4000 in 2007.

ALA can be applied topically to lesions to be treated or can be injected directly into them. Alternatively, the prodrug can be administered orally or parenterally. While each of these approaches leads to PpIX accumulation in the target cells, each is associated with significant difficulties. ALA penetration into deep lesions is unsatisfactory when applied topically and intralesional injection causes appreciable pain. Systemically-administered ALA causes systemic side effects and can only be given in limited doses.

Chemical modification of the parent ALA molecule is aimed at improving the efficiency of ALA-based PDT by increasing ALA delivery, enhancing PpIX accumulation and reducing side effects. This review examines the various chemical approaches taken to achieve these goals.

## Photodynamic Therapy

### Mechanism of action of photodynamic therapy

**Figure 1.**
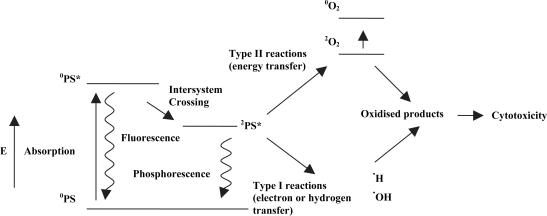
The mechanism of action of photodynamic therapy. Numbers in superscripts denote the number of unpaired electron spins in each molecule. Adapted from Konan et al. (2002).^10^

The detailed mechanism of action of PDT has been discussed extensively elsewhere.^9^–^11^ Briefly, it results from the interaction of photons of visible light, of appropriate wavelength, with intracellular concentrations of photosensitising molecules. Photosensitisers have a stable electronic configuration, which is in a singlet state in their lowest or ground energy level, °PS^10^ (Fig. 1). This means that there are no unpaired electron spins.^12^,^13^ Following absorption of a photon of light of specific wavelength, a molecule is promoted to an excited state, °PS*, which is also a singlet state and is short-lived with a half life between 10^−6^ and 10^−9^ seconds.^10^,^11^ The photosensitiser can return to the ground state by emitting a photon as light energy, or, in other words, by fluorescence, or by internal conversion with energy lost as heat. Alternatively, the molecule may convert to the triplet state, 2PS^*^. This conversion occurs *via* intersystem crossing, which involves a change in the spin of an electron.^14^ The triplet state photosensitiser has lower energy than the singlet state but has a longer lifetime.

The singlet state sensitiser can interact with surrounding molecules *via* Type I reactions, while the triplet state sensitiser can interact with its surroundings *via* Type II reactions. The former type of reaction leads to the production of free radicals or radical ions, *via* hydrogen or electron transfer. These reactive species, after interaction with oxygen, can produce highly reactive oxygen species, such as the superoxide and peroxide anions, which then attack cellular targets.^9^ However, Type I reactions do not necessarily require oxygen and can cause cellular damage directly, through the action of free radicals, which may include sensitiser radicals. Type II reactions, by contrast, require an energy transfer mechanism from the triplet-state sensitiser to molecular oxygen, which itself normally occupies the triplet ground state, 2O_2_^3^. Although possessing a short lifetime of approximately 10^−6^ seconds, a sufficient concentration of highly cytotoxic singlet oxygen, °O_2_, is produced to induce irreversible cell damage.^9^,^11^ In addition, the photosensitiser is not necessarily destroyed, but can return to its ground state by phosphorescence without chemical alteration and may be able to repeat the process of energy transfer many times.^14^ Alternatively, the sensitiser may return to ground by transferring its energy to molecular oxygen, and may even be destroyed by photobleaching due to oxidation.^15^ Evidently, many effects of PDT are oxygen-dependent and rely on the oxygen tension within the target tissue. Type I and Type II reactions can occur simultaneously and the ratio between the two depend on the photosensitiser, substrate, oxygen concentration and sensitiser to substrate binding.^9^ Singlet oxygen is, however, widely believed to be the major damaging species in PDT.^1^,^2^,^10^ Due to its extreme reactivity, singlet oxygen has a short lifespan in a cellular environment and limited diffusivity in tissue, allowing it to travel only approximately 0.1 μm.^16^ This, combined with the facts that normal tissue may not contain photosensitiser or may not be perfused by blood vessels damaged by PDT, means that damage to normal cells is minimal.^17^

### Photosensitisers

The ideal photosensitiser is one that shows a high tumour to normal tissue ratio, exhibits rapid accumulation in tumour tissue and is cleared efficiently from the body.^18^,^19^ Localisation of preformed photosensitisers in neoplastic tissue has been shown, though its mechanism is not completely understood. Preformed, lipophilic sensitisers, such as the porphyrins and phthalocyanines, when administered intravenously, are believed to be transported in the bloodstream bound to lipoproteins, such as low density lipoproteins (LDLs).^14^,^20^ Tumour cell membranes are known to possess disproportionately high numbers of LDL receptors,^21^ leading to active accumulation of photosensitiser molecules at close proximity to tumour cells. Photosensitisers may also accumulate in tumours due to abnormalities in the local microvasculature, including disordered a blood supply and enhanced vascular permeability.^20^,^22^

### 5-Aminolevulinic acid (ALA)

ALA is a small, water-soluble, prodrug that is a naturally occurring precursor in the biosynthetic pathway of haem. Administration of excess exogenous ALA avoids the negative feedback control that haem exerts over its biosynthetic pathway. Due to the limited capacity of ferrochelatase to convert PpIX into haem, the presence of excess exogenous ALA in cells induces accumulation of PpIX.^23^–^25^ This effect is pronounced in sebaceous glands and also in neoplastic cells. It has been reported that certain types of neoplastic cells have not only reduced ferrochelatase activity, but also enhanced porphobilinogen deaminase (PBGD) activity.^3^,^9^,^26^ Low ferrochelatase activity may be of lesser important to deficiencies in mitochondrial energy generation because tumour cells typically have low activities of mitochondrial cytochrome oxidase and utilise glycolysis rather than oxidative phosphorylation.^17^ In addition, certain malignant cells have low iron stores, a characteristic of proliferating cells, leading both to increased expression of transferrin receptors and, importantly, to decreased conversion of PpIX into haem.^27^,^28^ Porphobilinogen deaminase is considered to have the lowest activity in the haem biosynthetic pathway^29^,^30^ and the reason for its up-regulation in certain tumour cells has not yet been elucidated.^9^ It is generally considered to be rate-limiting in the ALA-induced synthesis of PpIX in neoplastic cells.^31^ Notwithstanding this, other reports have failed to find a clear relationship connection between high PBGD and/or low ferrochelatase activity and PpIX accumulation.^32^–^34^

### Clinical administration of ALA

To date, clinical applications of PDT have been limited to areas of the body easily amenable to irradiation from laser or incoherent light sources. Consequently, PDT has been primarily investigated as a treatment for tumours and neoplasias of the skin, bladder, mouth and female reproductive tract. Compounds of high molecular weight (>500 daltons) have inherently low permeabilities of the *stratum corneum* barrier of the skin.^35^ Therefore, notwithstanding a few isolated studies,^36^,^37^ pre-formed photosensitisers, which are generally large, highly conjugated molecules, are not commonly used in topical PDT. This, coupled with their inherent lack of selectivity, means that ALA, a photosensitiser prodrug with a relatively low molecular weight of 167.8 daltons, is the most frequently employed agent in modern topical PDT.

PDT, based on topical application of ALA, has been successfully used in the treatment of basal cell carcinoma,^23^,^38^,^39^ actinic keratosis,^24^,^40^,^41^ Bowen’s disease,^42^–^44^ vulval intraepithelial neoplasia,^45^–^47^ vulval Paget’s disease^48^ and cervical intraepithelial neoplasia.^49^ Due to the highly selective accumulation of PpIX in neoplastic cells resulting from topical application of ALA, the technique has also found use in the photodiagnosis (PDD) of neoplastic lesions of the mouth,^50^ bladder,^51^,^52^ endometrium^53^ and cervix.^54^ Illumination of the treated area with UV light causes reddish-pink PpIX fluorescence in neoplastic tissue, while the surrounding healthy tissue appears blue. The technique often allows detection of sub-clinical lesions, which may be missed by conventional means of examination.

PDT using topically applied ALA, in addition to producing successful therapeutic outcomes with excellent tissue preservation and no scarring, does not give rise to prolonged cutaneous phototoxicity.^38^,^55^ Thus, ALA-PDT can be repeated often without causing accumulation of PpIX in normal skin.^11^ This is particularly important when the aim of treatment is primarily palliative. This is in contrast to conventional PDT using the older preformed sensitisers, such as haematoporphyrin derivative. Repeated administration of such agents leads to persistent high photosensitiser levels in normal skin and severe phototoxic reactions after sun exposure.^10^,^23^

As ALA is a small molecule, its diffusion into cutaneous tissue from a topical delivery system should be efficient. However, the hydrophilic nature of ALA, as evident from its low octanol:water partition coefficient of 0.03,^56^ does impair permeation markedly, since skin presents an essentially hydrophobic barrier to the permeation of exogenously applied agents, As a result, topically applied ALA penetrates intact *stratum corneum* poorly, making it the principal barrier to effective absorption.^57^,^58^ Fortuitously, the disordered *stratum corneum* and disrupted epithelial barriers offered by many neoplastic lesions allow enhanced ALA penetration, due to poor continuity in intercellular lipid structures.^8^ This further improves the selectivity of PpIX accumulation and explains why ALA can be successfully employed for diagnostic purposes. However, its low lipophilicity^59^ does prevent effective penetration into hyperkeratotic lesions,^38^,^40^,^60^ and may even facilitate efflux, *via* the local micro-circulation, from deep nodular lesions.^31^

The clinical success of PDT relies on achieving a threshold concentration of ALA after topical application that induces therapeutic levels of protoporphyrin IX (PpIX) in abnormal cells. Failure to achieve this threshold leads to insufficient amounts of PpIX and irradiation of the target cells will then not generate enough singlet oxygen to eradicate the lesion successfully. Clearly, it is important that this threshold is evaluated. Cell culture experiments have demonstrated that concentrations of interstitial ALA must reach levels between 0.01 mg ml^−1^ and 0.17 mg ml^−1^ before sufficient PpIX is produced to cause a significant (>90% kill) level of neoplastic cell death upon illumination with an optimised dose of red light.^15^,^61^–^63^

The more common means of drug administration have been used for ALA delivery, such as the oral^64^,^65^ and parenteral^64^ routes. Although PpIX accumulation and prolonged patient photosensitisation are not problematic, ALA has been shown to be rapidly eliminated from the human body, with a plasma half-life of 50 minutes when given intravenously and 4.5 minutes when given orally.^66^ The small volume of distribution of only 8.3 L indicates that a large portion will be by broken down by first-pass metabolism or excreted unchanged in the urine. Studies involving dogs revealed that >50% of an administered drug dose will distribute into the liver and that approximately 15% will distribute into the kidneys.^67^ The pharmacokinetic profile of ALA is, therefore, highly unfavorable with respect to the generation of photodynamically efficient levels of PpIX after systemic administration. Apart from its limited bioavailability, systemic administration of ALA is associated with a number of side effects in humans. In addition to nausea, vomiting and transient abnormal liver functions, significant decreases in systolic and diastolic blood and pulmonary pressure have been reported^68^,^69^ side effects, such as nausea^70^ and abnormalities of liver function, are of concern.^71^,^72^ This means that systemic ALA doses are limited to an upper ceiling of 60 mg kg^−1^ in most cases. In addition, it has been reported that ALA, when given systemically, can permeate across the blood-brain barrier, but the clinical implications of this observation are still unclear.^73^ These factors ensure that the topical route remains a viable alternative, especially when the neoplastic lesion is superficial in nature. In such cases, the diffusion of ALA through the *stratum corneum* and its ability to reach deep sites becomes an important consideration. In a small number of studies, this barrier to ALA diffusion has been bypassed completely and the drug has been administered via painful intracutaneous injection directly into skin tumours.^74^,^75^ Generally, though, more conventional strategies to enhance ALA penetration into such lesions have been devised. These include physical methods, such as tape-stripping, curettage and iontophoresis and chemical methods, such as the use of penetration enhancers or, most commonly, chemical derivatives of ALA.^8^

Recently, numerous ALA derivatives have been synthesised by various groups Worldwide. The aim of this work has been improved bioavailability of both systemically- and topically-administered ALA. With respect to topically-applied ALA, lipophilic ALA derivatives have been produced in the hope of enhancing tissue penetration.

## Synthesis of ALA and its Derivatives

**Figure 2.**
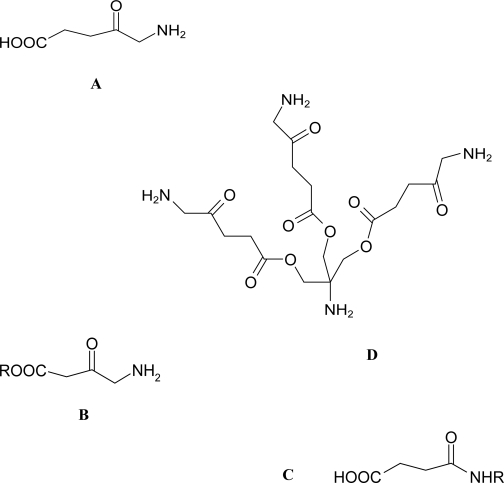
Chemical structure of 5-aminolevulinic acid (**A**) and general structures of its ester (**B**) and amide (**C**) derivatives and aminomethane tris-methyl ALA (**D**) which contains three ALA residues coupled *via* ester linkages to a central core. This type of dendron forms the building block for the 2nd generation 6-ALA and 3rd generation 18-ALA dendrimers.^115^

ALA is a straight chained δ-amino acid (Fig. 2). In living organisms there are two possible routes that have been reported for ALA synthesis.^76^ In the mitochondria of animals, yeast and fungi, ALA results from condensation of succinyl CoA and glycine, catalysed by ALA synthetase.^77^ Plants, algae and nearly all bacterial groups utilise the 5-carbon route to biosynthesis, where glutamate is converted to ALA along a three enzyme pathway.^78^ ALA can also be chemically synthesised from N-substituted amino acids and through stepwise build up of the carbon chain. Alternatively, amination of levulinc acid can also be employed.^79^

Numerous ALA derivatives of varying lipophilicities have been synthesised by reaction at either the amino group or carboxylic acid group (Fig. 2). ALA-containing dendrimers (Fig. 2), which are structurally defined hyper-branched polymers, have also been produced.

The most commonly-employed method to increase the lipophilicity of ALA has been to esterify the parent compound by reaction with an alkyl alcohol. ALA esters have typically been synthesised under standard conditions for esterification of acidic drug substances, employing the appropriate alcohol and thionylchloride or hydrochloric acid.^80^ By doing so, a large number of linear, branched, cyclic and ethylene glycol derivatives have been prepared. However, in some instances it may be of benefit to use tert-butylcar-bonyl (BOC) chemistry to prevent cross reactivity when using carbodiimide coupling to activate the carboxylic function. However, care should be taken about the acid used for deprotection of the BOC function, because the resulting salts (e.g. HCl or TFA) might substantially alter the pharmacokinetics of the active compound.^81^ A number of halogenated alcohol derivatives have also been synthesised that enhance lipophilicity further relative to their hydrocarbonyl counterparts. In this way, the lipophilicity of ALA can be modified over several orders of magnitude.^82^

The alternative approach to alter the lipophilicity of ALA using a simple derivatisation is to convert the aminogroup into an amide. In its most simple embodiment, this can be achieved by the condensation of ALA or one of its ester derivatives with acetic anhydride or acetylchloride under basic conditions.^80^

It is known that elevated levels of aminopeptidase activity exist in certain human tumour cell lines and in tumour-associated vasculature.^83^–^85^ The use of aminoacyl derivatives of ALA to address these altered enzymatic activities has been investigated experimentally by Berger et al.^86^ who prepared a number of BOC-protected and deprotected pseudopeptide derivatives of ALA and ALA esters and dipeptide derivatives of ALA.^87^ The aim was to utilise these compounds to further enhance the selectivity of PpIX accumulation in neoplastic cells rather than to enhance lipophilicity for enhanced percutaneous penetration.

One chemical strategy aimed at overcoming the poor systemic bioavailability of ALA has been the synthesis of structurally-defined ALA-containing dendrimers. In this way, a number of ALA molecules can be delivered by the same dendrimer. The minimal structure of a dendrimers is known as a dendron. Such dendron and dendrimers moieties exhibit the enhanced permeability and retention effect in tumours.^88^ This may enhance tumour-specific ALA delivery and lead to increased specificity of PpIX accumulation in tumour cells.

## Tissue Penetration of Topically-applied ALA and ALA-derivatives

Numerous literature reports describe both *in vitro* and *in vivo* penetration of topically applied ALA into tissue. However, while a range of techniques have been used to assess the final penetration depth, few report on the specific ALA concentrations found at various depths from the plane of surface absorption. Most studies to date have used fluorescence microscopy to investigate the formation of PpIX in tissue, after topical application of ALA. In the majority of cases, an ALA-containing vehicle is applied topically to normal or diseased skin of animal or human volunteers. The formulation is generally left in place for 4–6 hours before a biopsy is taken from the application site. Sectioning of the biopsy allows the fluorescence intensity of PpIX to be evaluated using suitable microscopy with excitation wavelengths around 400 nm and emission wavelengths from 600–700 nm.^3^,^88^ Alternatively, PpIX is extracted from dissolved tissue samples and determined using fluorescence spectrophotometry with similar excitation and emission wavelengths as above.^27^,^89^,^90^ It is clear that most studies tend to be qualitative in nature, comparing PpIX fluorescence with background auto-fluorescence. They serve, simply, to give an indication of depth of PpIX formation and, by inference, ALA penetration. Konig et al.^91^ reported PpIX fluorescence at a depth of 0.6 mm, 6 hours after topical ALA application, in patients with skin tumours. Szeimies et al.^92^ reported PpIX fluorescence at a depth of 0.3 mm in basal cell carcinomas (BCC). In contrast, Pahernik et al.^93^ reported PpIX fluorescence at depths as low as 3 mm in hamster skin tumour models.

Wennberg et al.^94^ used microdialysis to quantify ALA in normal skin and BCC after topical application of ALA (20% w/w) in an aqueous gel. A microdialysis tube was inserted intracutaneously at a depth of 0.5 mm and samples were taken at regular time intervals for analysis by high performance liquid chromatography (HPLC). The concentration of ALA in BCCs at a depth of 0.5 mm was found to range between 0 mg ml^−1^ and 0.52 mg ml^−1^. No ALA could be detected in normal skin at this depth. While this study was able to quantify ALA concentrations at a depth of 0.5 mm, the drug concentrations above and below this depth could not be assessed, nor could the depth of ALA penetration.

Casas et al.^57^ using liquid scintillation spectrometry, showed that ALA could penetrate model mouse tumours down to depths of 5 mm, although the majority of drug was found in the upper 2 mm of tissue. ALA concentrations at the different depths were not reported, in contrast to the studies carried out by Ahmadi et al.^95^ and McLoone et al.^96^ In these studies, the authors used liquid scintillation spectrometry to determine the concentrations of ALA at varying depths from the surface of nodular BCC. Concentrations of ALA as high as 20 mg ml^−1^ were detected at depths as low as 2 mm in these nodular lesions. However, once the integrity of the SC is more intact, as in normal skin, then permeation is shown to slow. Johnson et al.^97^ used autoradiography to demonstrate that the majority of a topically applied ALA dose did not penetrate porcine skin much deeper than the lower reaches of the *stratum corneum*. The same authors, using scintillation spectrometry, reported that ALA pasting through the entire *stratum corneum* barrier only achieved depths of 100–150 μm in underlying tissue. The importance of the *stratum corneum* as a barrier to ALA penetration was illustrated by Donnelly et al.^98^ The authors showed, using autoradiography, that topically applied ALA could penetrate vaginal tissue, which possesses no *stratum corneum* barrier, down to depths of at least 6 mm.

The lipophilicity and molecular weight of a drug substance are considered to be the primary determinants of diffusivity through *stratum corneum*.^99^ This layer of the epidermis comprises mostly anucleate cells and is associated with providing the principal barrier function in respect of transdermal delivery of drugs. The *stratum corneum* has been portrayed as the often cited ‘brick and mortar’ model in which keratinised cells are embedded in a mortar of lipid bilayers. The intercellular route of drug diffusion through the will not, therefore, be an accessible phase for either very polar or charged species.

Enhanced permeability through the lipid networks in the *stratum corneum* can be undertaken by chemical derivatisation of ALA.^100^,^101^ This pro-drug strategy imparts a greater lipophilicity to the parent ALA compound, usually accomplished by formation of a labile bond at the amino^86^,^87^ or, most commonly, the carboxylic acid group of the parent ALA molecule. Table 1 shows the octanol/water partition coefficients and the *stratum corneum*/water partition coefficients of ALA and several commonly employed ALA esters. It can be seen clearly that increasing the alkyl chain length of ALA esters significantly enhances lipophilicity and the ability to partition into the *stratum corneum*. However, within a homologous series, drug permeability usually increases with log *P*_oct_ up to a maximum, at which point transport becomes limited due to sequestration of the drug within the lipophilic barrier. Consequently, following topical application to skin, PpIX fluorescence induced by ALA esters is localised to the site of application, while fluorescence arising from ALA-induced PpIX is typically observed at distant locations. This effect may be directly attributable to pro-drug sequestration within the *stratum corneum*, whereas the more hydrophilic ALA is able to penetrate to vascular networks more effectively. However, since PpIX and ALA are completely cleared from the body within 24 hours of administration,^102^ prolonged and undesirable widespread cutaneous photosensitivity is not problematic.

Numerous *in vitro* and *in vivo* studies have been carried out to assess the ability of ALA esters to enhance penetration and PpIX production. The majority of *in vitro* investigations reveal that increased amounts of ALA esters, relative to the parent compound, only penetrate *stratum corneum* after prolonged application times, sometimes approaching 30 hours. Working within a framework of clinically relevant application times, such as 4 or 6 hours, no significant difference is observed in amounts of ALA or ALA-esters penetrating *stratum corneum*, regardless of ester alkyl chain length.^103^–^105^ The *in vivo* studies have typically investigated PpIX production in the skin of human volunteers^106^–^108^ or nude mice^88^,^101^,^109^,^110^ following topical application of ALA or one of its esters. Again, significant lag times are generally observed before PpIX fluorescence induced by ALA esters becomes greater than that induced by ALA. These observations are in contrast to those found in PDD of dysplasia and early bladder cancer, which showed that the ALA hexyl ester could not only reduce instillation times, but induce a 2-fold increase in fluorescence signals with a 20 times lower concentration of ALA hexyl ester compared to ALA.^52^ These findings were attributed to the different properties of the urothelial and *stratum corneum* permeability barriers.

To date, no studies have been carried out on tissue penetration of amide derivatives of ALA or, understandably, ALA dendrimers.

## Cellular Uptake and Metabolic Conversion of ALA and ALA-derivatives

The tetrapyrrolic structure of porphyrins means that ALA amides must be cleaved before entering the haem biosynthetic pathway. This hypothesis is supported by the experimental observation that ALA amides and their esters generally fail to induce large amounts of PpIX *in vitro*^80^,^86^,^111^ and *in vivo*^80^,^111^ in the absence of specific peptidases. Moreover, Moan et al.^112^ have shown that an N-formyl ALA derivative neither induced porphyrin synthesis nor inhibited the formation of PpIX induced by ALA. However, as discussed previously, these characteristics might be advantageously used to further increase the selectivity of ALA-induced PpIX accumulation by targeting specific proteases found in abundance in some tumors.^86^,^113^,^114^

It is well accepted that ALA-containing dendrimers and dendrons must release their cargo in order to allow PpIX production. However, this does not necessarily have to occur outside the cell, with intact dendrons having been detected within cells *in vitro*.^115^ Dendrimers containing up to 18 ALA residues have been prepared and used to substantially improve PpIX production in cells relative to ALA.^116^ Experiments performed with ALA-containing dendrimers and dendrons have generally produced mixed results, however 115–117. Release from the ALA-containing structure may be problematic, as evidenced by the fact that two thirds of intracellular tetrameric ALA-containing dendrons remained intact 3 hours after administration.^115^

Thus, ALA amides must be cleaved and ALA-containing dendrimers must also be able to efficiently release ALA in order to be potent substrates for PpIX production.

ALA-induced formation of PpIX depends on the penetration of ALA through the cell membrane. Being a zwitterion with pK_a_ values of 4 (carboxylic acid group) and 8.9 (amino group),^57^ the lipophilicity of ALA is unlikely to change significantly in the physiological pH range. It is expected, therefore, that ALA is unlikely to enter cells by passive diffusion alone. In *Salmonella typhimurium*^118^ and *Escherichia coli*^119^ the dipeptide permease is probably responsible for ALA transport across the cell membrane. In eukaryotic cells, the uptake mechanisms are not clear. ALA uptake may rely on an active transport mechanism, as exemplified by that in *Saccharomyces cerevisieae*, which shows an apparent K_m_ of 0.1 mM at an optimum extracellular pH of 5.0.^120^ Mammalian cells may possess additional cell-type dependent mechanisms,^9^ since ALA uptake in rat cerebellum particles, for example, was found to be nonsaturable up to 4 mM of ALA.^62^ In addition, Rud et al.^121^ showed that ALA is transported into human adenocarcinoma cells by β–amino acid and γ–aminobutyric acid carriers and is Na^+^ and partly Cl^−^ dependent. The PEPT1 and PEPT2 transporters have also been identified as potential transporter systems for ALA uptake.^122^,^123^

The methyl ester of ALA has been shown to be taken up actively by WiDr cells using transporters of non-polar amino acids.^124^ However, longer chain aliphatic ALA esters are not transported by these carriers and it has been postulated that they may enter cells by either passive diffusion or endocytosis.^125^,^126^ Once in the cell, the esters may be converted to ALA by non-specific esterases. Alternatively, the esters may be hydrolysed to ALA outside the cell. Indeed the skin, in particular, possesses a multitude of different enzymes by which topically applied drugs can be metabolized.^127^ However, it has yet to be demonstrated conclusively that short chain ALA esters actually need to be hydrolysed in order to enter the haem biosynthetic pathway.^128^ While it is accepted that stearically-hindered, or extremely large ALA esters would be unable to act as substrates for porphobilinogen synthetase, which converts two ALA molecules into porphobilinogen (Janet et al. 2000), short chain esters may be able to produce PpIX esters, which are likely to have very similar photochemical properties to PpIX.

Cell culture studies have demonstrated that aliphatic straight chain ALA esters, up as far as the hexyl ester in the homologous series, induce higher levels of PpIX in neoplastic cells more rapidly than the parent compound,^62^,^63^,^80^,^82^,^109^,^129^,^130^ presumably due to their non-requirement for a saturable active transport mechanism. The optimum PpIX fluorescence in intact murine mammary cancer cell spheroids (275–350 μm) was shown using 0.05 mM hexyl ALA, almost 200 times lower than the optimum concentration of ALA (10 mM). This indicated that not only did the interior cells maintain esterase activity and porphyrin synthesis, but that hexyl ALA diffused efficiently to the spheroid interior.^131^

It has been suggested that lower concentrations of ALA esters, with shorter application times would increase the efficacy of PDT and PDD.^125^ However, with the exception of the case of bladder instillation, topical application of ALA esters does not seem to provide such an advantage. The results obtained from *in vitro* cell culture studies demonstrate that the time required for cleavage of the ester group to yield free ALA appears to be insignificant and does not seem to limit the usefulness of ALA esters. Retention in, and gradual release from, the *stratum corneum*, as discussed above, in combination with poor release from the topically applied vehicle may, therefore, be mostly responsible for the observed lag times before significant *in vivo* PpIX production. In spite of this, ALA methyl ester has been shown to be effective for PDT of nodular basal cell carcinoma^59^,^132^,^133^ where ALA PDT has historically produced poor results.^134^,^135^ However, it should be pointed out that these clinical studies used curettage/debulking to remove the *stratum corneum* and some of the carcinoma before treatment, and also routinely used a 1–2 treatment cycles that each involved two treatments a week apart. Nevertheless, a topical cream containing 16% w/w methyl ALA (Metvix^®^, Photocure, Norway) has received market authorisation in the US and Europe.

## Stability of ALA and its Derivatives

**Figure 3.**
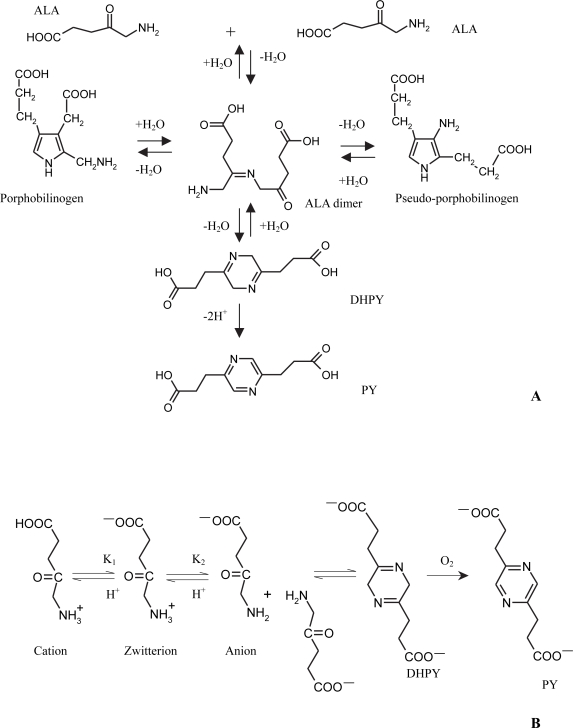
Possible condensation reactions involving 5-aminolevulinic acid (**A**) and pH-dependent equilibria occurring in aqueous solutions of 5-aminolevulinic acid (**B**) Adapted from Novo et al. (1996).^51^

ALA belongs to the class of α-aminoketones, which dimerise readily under alkaline conditions.^136^ The formed dihydropyrazines can further oxidise to pyrazines. Formulation of ALA into drug delivery systems for photodynamic therapy has necessitated the derivation of its degradation pathways. Under alkaline conditions, the formation of 3,6-dihydropyrazine 2,5-dipropionic acid (DHPY), porphobilinogen and pseudo-porphobilinogen, via an open-chain dimeric ketimine, from ALA has been postulated previously. Furthermore, the oxidation of DHPY to pyrazine 2,5-dipropionic acid (PY) has also been reported. The possible condensation products of ALA, formed under alkaline conditions and in the absence of enzymes, according to the literature, are shown in Figure. 3(A)^137^–^141^ Dalton et al. 1999. In addition to the cyclic degradation products, it has also been suggested that ALA may undergo a polymerisation reaction in solution.^142^

Initially, the published reports on ALA degradation mechanisms and products were conflicting. This conflict can be ascribed to the variety of stability test conditions and analytical methods used. Most studies did not elucidate the structure of the degradation products. However, Bunke et al.^143^ using capillary electrophoresis (CE) and nuclear magnetic resonance spectroscopy (NMR), have shown that only two condensation products for ALA exist in alkaline media in the absence of enzymes. DHPY is formed initially and this is then oxidised to PY, which is the major degradation product in aerated solutions. Neither porphobilinogen nor pseudo-porphobilinogen are formed under such conditions. Novo et al.^51^ De Blois et al.^144^ and Gadmar et al.^142^ using ultraviolet spectroscopic methods, have come to similar conclusions.

The stability of 5-aminolevulinic acid (ALA) in aqueous solution has been shown to be dependent on four factors, namely, pH, concentration, temperature and degree of oxygenation of the solution.^51^,^142^,^144^,^145^

It has been proposed that 2 molecules of ALA can only react to form 3,6-dihydropyrazine 2,5-dipropionic acid (DHPY), when the amino group of the ALA is deprotonated. The DHPY is then oxidised to pyrazine 2,5-dipropionic acid in aerated media.^51^ The pH dependence of the reaction of ALA can, thus, be explained on the basis of the acid-base equilibria of this amino acid,^146^ which is shown diagrammatically in Figure. 3(B).

The values of the acid dissociation constants (pK_a_) of ALA have been shown to be around pK_1_ = 4.0 and pK_2_ = 8.3.^51^,^147^ These values indicate that the zwitterion is the major species present in the pH range between 5 and 7.5, although significant amounts of the two other acid-base species exist, depending on the acidity. Therefore, at pH 5.0, about 10% of the ALA molecules are cations, whereas around pH 7.3, for example, about 10% of the ALA molecules are anions. Accordingly, a scheme similar to that proposed by Butler and George^140^ could explain the reaction involving ALA. The anion, a species with a deprotonated amino group, is the only one able to react with the ketone group of a neighbouring molecule to yield the cyclic dihydropyrazine, DHPY. For the condensation to occur, the amino group of ALA should be deprotonated. This explains the strong pH dependence of the reaction, since the concentration of the anion increases with the pH. Therefore, ALA solutions are only stable at low pH values, where the anion does not exist.

The theories discussed above have been investigated experimentally by a number of workers. De Blois et al.^144^ showed that ALA solutions, of an initial concentration of 0.1% w/w and pH values of pH 4.0, were still within the pharmaceutically acceptable range of 90–100% w/w ALA after 128 days storage at 21 °C. At pH 8.0 the ALA content declined below the 90% w/w limit within a few days. Elfsson et al.^147^ found that solutions of ALA, buffered to pH 2.35 were completely stable over a period of 37 days, even when stored at 50 °C. The half lives for the decomposition of ALA at pH 4.81 and pH 7, respectively, at 50 °C were 257 hours and 3 hours, respectively. Novo et al.^51^ showed that a 0.3 M solution of ALA in distilled water had a pH of 2 and was completely stable under various conditions of storage. All three groups reported decrease in pH in degrading solutions of ALA. This may be explained by the fact that as the anion reacts, the concentration of protons increases in the solutions of ALA in order to maintain the zwitterion-anion equilibrium.^51^,^144^

The degradation of ALA has been shown experimentally to follow second order kinetics.^144^,^145^ Hence, doubling the concentration of ALA in a solution of a given temperature and pH should quadruple its rate of decomposition. De Blois et al.^144^ showed that a solution at pH 5.0, with an initial ALA concentration of 0.5% w/w, had ALA concentrations that were still higher than 90% w/w after 178 days storage. Solutions with initial ALA concentrations of 2, 5 and 10% w/w, had ALA contents which dropped below the 90% limit after 150, 94 and 29 days, respectively. Elfsson et al.^147^ used Arrhenius plots to interpret the results from accelerated storage testing of ALA solutions. The authors showed that a 1% w/w solution of ALA, stored at pH 7.53, would have a shelf life, or t_90_, the time it takes for a substance to lose 10% of its initial mass, of 1.9 hours at 20 °C. The shelf life (t90) of a 10% w/w solution of ALA at pH 7.53 would be as short as 10 minutes at 20 °C.

It is well known that, in general, as the concentration of a reactant is increased, the rate of reaction increases. Elfsson et al.^147^ incubated 1% w/w solutions of ALA at pH 7.53 and at temperatures ranging from 37 °C to 85 °C. It was shown that, for every 10 °C rise in temperature, the rate of ALA degradation increased by a factor of around 1.5.

The initial second order degradation of ALA to give DHPY has been shown to be reversible by acidification of the solution, providing no oxygen is present in the reaction mixture^145^ If oxygen is present then the DHPY is irreversibly converted to PY and the ALA lost is irretrievable.

During degradation, aqueous solutions of ALA are reported to undergo colour changes over time, changing from colourless to yellow and then to red/orange.^144^,^145^ The yellow colouration is attributed to DHPY, based on UV studies of deaereated solutions,^51^ while the red colour is attributed to PY.^145^

The above considerations have led to the development of various strategies for maintaining the stability of ALA in solution. Neither addition of ethylene diamine tetra acetic acid (EDTA)^142^,^147^ nor antioxidants^145^ to ALA solutions was able to prevent degradation. Most workers have advised dissolving ALA in solutions buffered to around pH 2.0 to maintain long-term stability. Due to the potential for cutaneous irritancy at such pH values, ALA solutions, buffered to physiological pH values, such as pH 5.5 or pH 7.4, are normally prepared immediately prior to use.

The majority of studies on ALA stability have investigated degradation in simple aqueous solution or in drug delivery systems. However, a number of studies have shown that PY can also be formed readily under *in vivo* conditions.^141^,^148^ Given that this compound has unknown toxicity, its formation in drug delivery systems and *in vivo* should be kept to a minimum.

Little published data exists on stability of ALA derivatives. Data on hydrolysis of ALA esters under various *in vitro* conditions even appears to be scarce, though the hexyl ester seems quite resistant to hydrolysis.^149^ All ALA esters have the potential to be hydrolysed *in vivo* by, for example non specific esterases in the skin or blood. Indeed, the esters may form pyrazine-type degradation products *in vitro* and *in vivo* before or after hydrolysis. However, we have previously shown^150^ that increasing the ester chain length can substantially reduce formation of pyrazine-type degradation products. ALA amides are likely to possess good stability profiles, as dimerisation will be prevented by stearic hindrance at the amino group. Similarly, ALA locked into dendron or dendrimers structures will be unable to react to produce degradation products, as they are not free to move.

## Drug Delivery Considerations

ALA and its derivatives are, in most cases, intended for topical application. However, as we have highlighted previously,^8^,^151^ despite the vast number of studies published in this area, a rational approach to formulation design has not taken place. When formulating a topical drug delivery system the aims should be to maximise the thermodynamic activity of the drug substance in the vehicle, so as to maximise the concentration drive for diffusion and maximise the partition coefficient between *stratum corneum* and vehicle. For example, formulating a relatively lipophilic ALA derivative, such as the hexyl ester in an aqueous vehicle should maximise its flux into skin when applied topically.

Studies published to date on topical application of ALA and its derivatives have used aqueous solutions, oil in water creams, water in oil creams, hydrogels, organogels, aqueous and solvent-based patches and particulate delivery systems. These dosage forms, which in many cases seem to have been selected at random with little regard to their nature, possess a multitude of different physico-chemical properties. This has made comparison of different studies difficult. As a result, the true value of derivatisation of ALA to yield more lipophilic prodrugs has been blurred somewhat. One example of such problematic comparisons lies in the iontophoretic delivery of ALA esters. ALA esters have a net positive charge at physiological pH. Because of this, electrorepulsion can be used to enhance the delivery of methyl ALA over and above that of ALA by approximately 50 times.^107^,^152^ The magnitude of this effect gradually decreased with increasing chain length within a homologous series of ALA esters. Conversely, Gerscher et al.^106^ could detect no significant difference between the levels of PpIX induced *in vivo* after iontophoresis of solutions of ALA, butyl ALA or hexyl ALA. This may have resulted from the acidic solutions used *in vivo*, which converted a greater fraction of ALA to the cationic form and elevated hydronium concentration. The latter carries charge more efficiently than ALA esters, and reversed the direction of electroosmotic flow.^125^

In order to uncover the true potential of ALA derivatives, a systematic formulation/skin penetration study is required. ALA derivatives (ester or amide) of increasing lipophilicites should be incorporated at defined concentrations into drug delivery vehicles, themselves of variable lipophilic character. The penetration characteristics of these drug/vehicle combinations should then be studied using excised skin, with the drugs quantified by HPLC or other sensitive analytical method.

## Conclusion

Chemical derivatives of 5-aminolevulinic (ALA) have the potential to improve bioavailability, enhance stability and lead to better therapeutic outcomes for treated patients. However, despite extensive recent investigation, ALA derivatives have yet to demonstrate meaningful clinical benefits, with the use of hexyl ALA for photodiagnosis of bladder neoplasias a notable exception. A rational approach to topical formulation design is required, along with a systematic study aimed at uncovering the true potential of ALA derivatives in photodynamic therapy. With respect to systemic ALA delivery, more study is required in the area of ALA-containing dendrons and dendrimers, with the aim being to enhance the selectivity and efficiency of ALA delivery and PpIX production while reducing systemic side effects.

## Figures and Tables

**Table 1.** Log *P*_oct_ and log *P*_*SC*/W_ for ALA and its ester derivatives.

**Compound‡**	**Mol. Wt. (Da)**	log*P*_oct_*	**log*P*_SC/W_†**
ALA	167.6	−1.5	−1.4
Methyl-ALA	181.6	−0.9	0.2
Butyl-ALA	223.8	1.4	0.3
Hexyl-ALA	251.8	1.8	0.9
Octyl-ALA	279.6	2.6	1.0

* *P* _oct_ is the partition coefficient between octanol and aqueous buffer solution (pH 7.4, 21 °C), as calculated by Uehlinger et al. (2000).^82^

^†^ *P_SC/_*_W_ is the partition coefficient between stratum corneum and water, as calculated by De Rosa et al. (2003).^104^

^‡^ hydrochloride salt (HCl.H_2_N-CH_2_-CO-CH_2_-COOR^1^ = general structure).
